# Cultural shifts

**DOI:** 10.7554/eLife.37432

**Published:** 2018-04-23

**Authors:** Bridget M Kuehn

**Affiliations:** eLifeChicagoUnited States

**Keywords:** scientist and parent, women in science, careers in science, diversity, mobility

## Abstract

China’s one-child policy has resulted in scientists with families having experiences that are very different to those elsewhere in the world.

China currently produces more scientific publications than any other country in the world. Economic reforms that began in the late 1970s, and a huge expansion of higher education that started in 1999, have fueled the rapid growth of Chinese science, according to a study by Chunni Zhang, a sociologist at Peking University, and co-workers. This expansion, coupled with dramatic shifts in Chinese society over the past three decades, has created new opportunities and challenges for female scientists in China, particularly those with children.

Female students outnumber men on science courses at some top universities. One of the factors that has driven women’s participation was the ‘one-child policy’ that was introduced in 1979 and lasted until it was replaced by a ‘two-child policy’ in 2016. Families wanted their child to be educated, whether it was a boy or a girl, so women’s education caught up with men’s. Furthermore, “Chinese parents think that a science major is good,” adds Zhang.

## Changing times and countries

Ling-An Wu, an emeritus professor of physics at the Chinese Academy of Sciences in Beijing, has lived through many of these changes. Her two sons were born just before the one-child policy came into effect, when good childcare centers were free and plentiful. This allowed her to work as an English translator and interpreter. “I was really lucky in that respect,” she says. Wu also benefited from a supportive husband who, like many men in the urban areas of China, shared the household duties.

The ‘Cultural Revolution’ had previously put her studies on hold but in 1981 at the age of 37, she was given the chance to pursue graduate studies in the US. With support from her mother and a nanny, who helped to look after her children who stayed in China, Wu was able to complete a PhD at the University of Texas at Austin. “It was my mother who said I should go to make up for what I lost during the Cultural Revolution,” recalls Wu. “I am really grateful to her.”

Now, younger Chinese women are grappling with a new set of challenges and opportunities. Like Wu, Nan Tang, an assistant investigator at the National Institute of Biological Sciences (NIBS) in Beijing, spent time in the US as a scientist parent. Her son Haikun Du was born when she was a postdoc working on embryonic lung development at the University of California, San Francisco. Many of her peers at UCSF were also having children so she was able to swap stories with them about getting pregnant or caring for a newborn.

“It was very, very supportive,” she says. That support helped to lessen her worries that having a child would slow down her career and got her through a tough bout of morning sickness during her first four months of pregnancy. Having health insurance through the university also proved crucial when she experienced a complication during delivery: “The medical bill was huge.”

Tang and her husband decided on a childcare center near his office, which meant that she spent more than an hour each way commuting to and from work on public transport every day. But she quickly learned to make the most of this time, using it to read and deal with emails. This allowed her to focus on her bench work and collaborations while she was in the office.

“I found I was more efficient at work,” says Tang. She also received a lot of support from her husband, who worked for a technology company: “Not only for science, but for every career you need support from the other side. I support him and at the same time he supports me.”

## On the baby bus

Tang moved back to China for her current position at NIBS in 2012. The institute recruits many junior principal investigators from the United States and other countries, and has strong systems in place for supporting parents. “They try to make the transition as smooth as they can,” she says. For example, the institute rented an apartment for Tang and her family before they arrived, and it helped to get her son enrolled in an elementary school.

Tang and her husband chose to send her son to an elementary school close to her husband's work. Most investigators send their children to another elementary school that is about six miles away from the institute. Children who attend this school ride what investigators affectionately refer to as the ‘baby shuttle’. Every morning the shuttle driver picks up the children from home and drives them to school; then, in the afternoon, he drives them from school to the institute so that they can go home with their parents.

**Figure 1:**
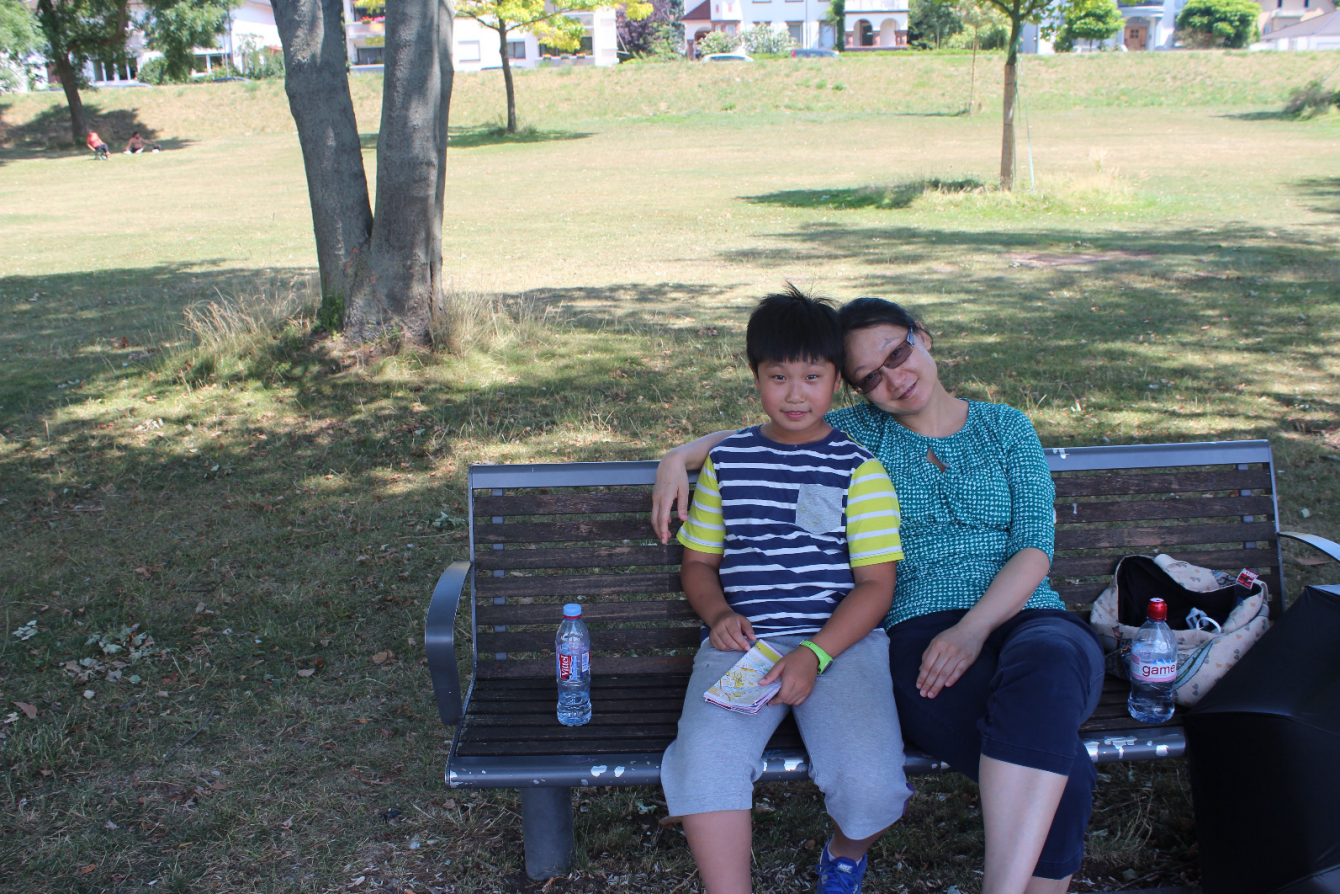
Nan Tang, shown here with her son Haikun Du, says that being a scientist and a parent requires hard work and organization, but is “well worth it.”

IMAGE CREDIT: Nan Tang.

All women in China are entitled to at least 98 days of maternity leave, and some employers offer more: Chunni Zhang, for example, has just finished a four-month maternity leave from Peking University. However, after the mother has returned to work it can be challenging to find childcare for children who are too young to go to school. In the past new parents often relied on grandparents for childcare, but many new parents – especially academics – now live far from their families. “This puts a lot of pressure on young couples,” says Zhang, particularly because the demand for childcare centers has exceeded supply since the one-child policy was relaxed. Ling-An Wu agrees that more childcare centres are needed.

Wu has experience enacting change. In 2000, after she had established herself as a researcher in China, she joined an international working group on women in physics. She was shocked to find that the number of women in physics in China was declining, particularly at the senior level. “I thought men and women had equal opportunities,” Wu says. “It was true for decades.”

She and the working group lobbied the National Natural Science Foundation, one of the biggest funding agencies in China, to extend the age limit for women applying for certain grants in physics from 35 to 38 to account for career breaks to raise children. She was pleasantly surprised when the foundation agreed to raise the age limit to 40 for women in all science disciplines. “It takes time to change the mindset, particularly of men,” says Wu. “You have to convince them there is a problem.”

## Sharing in science

Being a parent and a scientist requires hard work and organization, but Nan Tang thinks it is well worth it. She sometimes gets to teach her nine-year-old son and his classmates through a volunteer program at school. She also gets to share in his growing love of science, which includes dinosaurs and astronauts.

“Being a parent is the best thing in the world but being a scientist also is the best thing, I love my work,” Tang says. “It is so much fun to be a scientist.”

## Note

This Feature Article is part of the Scientist and Parent collection.

